# Compartmentalization Role of A-Kinase Anchoring Proteins (AKAPs) in Mediating Protein Kinase A (PKA) Signaling and Cardiomyocyte Hypertrophy

**DOI:** 10.3390/ijms16010218

**Published:** 2014-12-24

**Authors:** Abeer Rababa’h, Sonal Singh, Santosh V. Suryavanshi, Salah Eldien Altarabsheh, Salil V. Deo, Bradley K. McConnell

**Affiliations:** 1 Department of Clinical Pharmacy, Jordan University of Science and Technology, Irbid 22110, Jordan; 2 Department of Pharmacological and Pharmaceutical Sciences, University of Houston, Texas Medical Center, Houston, TX 77204, USA; E-Mails: srsingh@uh.edu (S.S.); svsuryavanshi@uh.edu (S.V.S.); 3 Department of Cardiac Surgery, Queen Alia Heart Institute, Amman 11953, Jordan; E-Mail: Salah936@yahoo.com; 4 Department of Cardiovascular Surgery, Case Western Reserve University, Cleveland, OH 44106, USA; E-Mail: Salildeo@yahoo.co.in

**Keywords:** hypertrophy, protein kinase A (PKA), A kinase anchoring protein (AKAP), contractility

## Abstract

The Beta-adrenergic receptors (β-ARs) stimulation enhances contractility through protein kinase-A (PKA) substrate phosphorylation. This PKA signaling is conferred in part by PKA binding to A-kinase anchoring proteins (AKAPs). AKAPs coordinate multi-protein signaling networks that are targeted to specific intracellular locations, resulting in the localization of enzyme activity and transmitting intracellular actions of neurotransmitters and hormones to its target substrates. In particular, mAKAP (muscle-selective AKAP) has been shown to be present on the nuclear envelope of cardiomyocytes with various proteins including: PKA-regulatory subunit (RIIα), phosphodiesterase-4D3, protein phosphatase-2A, and ryanodine receptor (RyR2). Therefore, through the coordination of spatial-temporal signaling of proteins and enzymes, mAKAP controls cyclic-adenosine monophosphate (cAMP) levels very tightly and functions as a regulator of PKA-mediated substrate phosphorylation leading to changes in calcium availability and myofilament calcium sensitivity. The goal of this review is to elucidate the critical compartmentalization role of mAKAP in mediating PKA signaling and regulating cardiomyocyte hypertrophy by acting as a scaffolding protein. Based on our literature search and studying the structure–function relationship between AKAP scaffolding protein and its binding partners, we propose possible explanations for the mechanism by which mAKAP promotes cardiac hypertrophy.

## 1. Introduction

The cAMP-dependent protein kinase A (PKA) is a threonine/serine kinase that has wide substrate specificity [1,2]. Similar to other threonine/serine kinases, PKA catalyzes the transfer of the γ-phosphate of ATP to threonine or serine residues, which are embedded in R-R-Ø-S motif (where Ø represents hydrophobic residues). The kinase is a heterotetramer consisting of two catalytic (C) and two regulatory (R) subunits. Each regulatory subunit is capable of binding two cAMP molecules. Once they bind to cAMP, specific conformational changes occur in the regulatory subunits, which lead to the activation and release of catalytic subunits. The freed catalytic subunits then disperse to phosphorylate PKA’s substrates [3].

The PKA substrate phosphorylation is required for many cellular processes including metabolism, gene expression and contraction [4]. In the heart, β-AR signaling activates PKA-RIIα, which phosphorylates many of the components involved in the excitation-coupling mechanism such as phospholamban (PLB), cardiac troponin I (cTnI), L-type calcium channel, cardiac myosin binding protein C (cMyBPC), phosphodiesterase 4D3 (PDE4D3), cAMP-responsive element binding protein (CREB) and the ryanodine receptor (RyR2) to amend their function and/or activity [5,6,7]. PKA-dependent phosphorylation of these and other related substrates increases the Ca^2+^ current, sarcoplasmic reticulum (SR) Ca^2+^ uptake and release, SR Ca^2+^ content, and the dissociation of Ca^2+^ from the myofilaments to facilitate the contraction and relaxation of the heart [8,9].

To facilitate PKA mediated signaling pathways, PKA is localized to distinct intracellular compartments by A-kinase anchoring proteins (AKAPs) that help in the specificity and efficiency of PKA signaling [10,11]. The role of PKA compartmentalization to specific intracellular compartments raised a critical role of AKAPs in complex networking performance through coordinating spatial-temporal signaling of proteins and enzymes that are regulated by cAMP second messenger.

## 2. A Kinase Anchoring Proteins (AKAPs)

The β-AR mediated activation of PKA by cAMP is the primary modulator for several cellular functions in the heart. These include cardiac contractility, ion fluxes, metabolism and even gene expression. Disturbances in the cAMP-mediated signaling can lead to cardiac hypertrophy and heart failure. It has been shown that a general increase in cAMP level is not enough for the specific regulation of proteins [12]. For instance, co-localization of PKA into a close proximity to its substrates is required to ensure spatial-temporal regulation of cAMP/PKA dependent substrates phosphorylation. PKA is localized to discrete intracellular compartments by AKAPs that help in increasing the specificity and efficiency of PKA signaling [10,11].

At present, over 70 different AKAPs have been identified in various cell types. AKAPs are diverse proteins that play a common dual role: (1) they behave as a scaffolding protein, to localize PKA to a special subcellular compartment; and (2) they enhance signal transduction through substrate phosphorylation. This co-localization decreases the time required for PKA substrate phosphorylation and also regulates which substrates are phosphorylated by scaffolding other signaling enzymes [13]. Via this spatial-temporal signaling of proteins and enzymes, regulated by second messenger system, AKAPs control substrate phosphorylation facilitating a unique signal regulation. Although AKAPs show no obvious sequence homology, all AKAPs share three major properties: (1) PKA anchoring domain, a conserved amphipathic helix of 14–18 amino acids, that interacts with the *N*-terminal dimerization and docking (D/D) hydrophobic groove of the PKA-regulatory subunits; (2) unique targeting domain positioning the AKAP complex in close proximity to its substrates and (3) binding sites for other signaling molecules [14,15,16,17,18].

Four PKA regulatory subunits have been identified (RIα/β and RIIα/β). The majority of type I PKA holoenzyme (RI α and β) is preferentially present in the cytosol [19], whereas all PKA-RII is predominantly targeted to specific subcellular sites by its interaction with AKAPs [14,20,21]. The PKA-RII subunits bind to AKAPs with nanomolar affinity, and by contrast, PKA-RI subunits bind to AKAPs with only micromolar affinity [14]. Most AKAPs bind to the PKA-RII subunits but some AKAPs still have dual specificity and bind RI subunits as well. The crystal structure of the PKA-RIIα D/D domain interacting with D-AKAP2 provides insight into how RIIα tethers a wide range of AKAPs with high affinity [22]. The first 30 amino acids residue of PKA-RII are necessary for AKAP binding as well as for dimerization of PKA-RII. Although, the RII dimerization is required for AKAP binding, both dimerization and binding AKAPs have separable regions. Additionally, autophosphorylation of the PKA-RII subunits result in an increase in the PKA/AKAP interaction [23]. Generally, The PKA/AKAP complex formation is a result of different protein and lipid interactions that occur through the unique targeting domains located within each AKAP [14].

Finally, AKAPs possess the ability to coordinate multiple signaling pathways by binding to different phosphatases and/or PDEs, which terminate the PKA signaling. AKAPs can also facilitate the integration of PKA signaling with other signaling pathways by containing signaling proteins of different pathways in their scaffold. Hence, AKAPs form a framework complex that synchronizes multi-protein signaling.

### 2.1. AKAPs in Cardiomyocytes

More than 14 different AKAPs have been shown to be expressed in both rodent and human heart tissue including: gravin (AKAP12), AKAP 15/18, mAKAP and yotiao. Each of these AKAPs localizes PKA to a different subcellular compartment, permitting a higher degree of selectivity and specificity of phosphorylation for different downstream PKA substrates. Furthermore, several AKAPs are known to be involved in modulation of cardiac contraction coupling. They help in generating the cardiac action potential and Ca^2+^handling in cardiomyocytes [24]. For example, AKAP18α is associated with the alpha subunit of L-type Ca^2+^ channel (Ca_(V)_1.2) and mediates PKA phosphorylation of the channel and reported to enhance channel activity as well as increased Ca^2+^current [25]. Of note, the splice variants of AKAP15/18 are now referred to as AKAP7 short and long isoforms [26], where the smaller AKAP7 isoforms (α and β) are expected to behave similarly and the longer AKAP7 isoforms (γ and δ) are expected to behave similarly [27]. Although it has been suggested that the short and long AKAP7 isoforms localize PKA complexes with Ca_(V)_1.2 and PLB, respectively, a recent report by Jones *et al.* [27] has found that AKAP7 is not required for regulation of Ca^2+^ handling in cardiomyocytes in response to adrenergic stimulation. However, AKAP79 can also cause the phosphorylation of L-type Ca^2+^channels and it is assumed that AKAP79 can substitute for AKAP7 (AKAP18α) [28].

Previous reports show that the muscle-selective AKAP (mAKAP, AKAP100, AKAP6) associates with RyR2 channels of the SR [29,30], and is involved in modulating the activity of RyR2 channel to facilitate Ca^2+^ induced Ca^2+^ release (CICR) from the SR for cardiac contraction. Recent data argue and confirm that mAKAP is primarily present on the nuclear envelope of cardiomyocytes. This specific binding is conferred via integral nuclear membrane protein, nesprin1α to which mAKAP is tethered by spectrin repeats [2,31,32,33]. mAKAP is also shown to be present at the Z-disc of cardiomyocytes and plays crucial role in intracellular transport of myopodin [34]. Gravin or AKAP250 which is expressed in the heart is involved in scaffolding and targeting PKA and PKC to β_2_-AR and is involved in the modulation of the desensitization and resensitization cycle of β_2_-ARs. We have shown that disruption of gravin’s scaffolding increases baseline contractility as well as augments contractility in response to acute β-AR stimulation [35].

The importance of PKA–AKAP interaction in regulating cardiac function has been established by studying the Ht31 peptide. This molecule competes with AKAPs to associate with PKA-RII subunits. The utilization of Ht31 peptide in cardiomyocytes results in the amended phosphorylation of PKA substrates including cTnI and cMyBPC upon β-AR activation with the agonist isoproterenol (ISO) [36]. Furthermore, Ht31 disruption of PKA/AKAP interactions resulted in decreased phosphorylation of PLB and the cardiac RyR2 channel. However, despite these changes in PKA substrate phosphorylation, contractility was improved in the presence of β-AR stimulation [37]. Additionally, disruption of particular PKA–AKAP interactions has also been shown to change β-AR interceded cardiac function. Another example for the importance of PKA/AKAP anchoring in regulating cardiac function is the disruption of yotiao’s interaction with KCNQ1, a voltage-gated potassium channel, which has been shown to be associated with the development of Long-QT syndrome. This syndrome is a chronic disorder characterized by prolonged repolarization of the action potential, which can lead to arrhythmia and sudden cardiac death [38]. Data from our laboratory have shown that mutations or SNPs in mAKAP can lead to significant alterations in the binding properties of mAKAP with its binding partners. We have identified some of these SNPs in our laboratory and have studied the changes these mutations can have in the binding affinities of mAKAP. One such SNP, mAKAP-S2195F, a mutation located in mAKAP-PP2A binding site, showed significant increase in both binding propensity to PKA as well as PKA-activity [39]. Hence, alterations in PKA/AKAP interactions can have reflective effects on β-AR signaling and cardiac performance.

### 2.2. Muscle Selective AKAPs (mAKAP, AKAP100, AKAP6)

The scaffolding protein muscle selective AKAP (mAKAP) is a 250 kDa PKA-anchoring partner that has been found to be expressed in the brain and heart. Two alternatively spliced forms of mAKAP are expressed: α and β [40]. The longer form mAKAPα is preferentially expressed in the brain. However, mAKAPβ, which is the shorter form of the scaffolding protein that lacks the first 244 amino acids, was found to be preferentially expressed in the heart at the nuclear envelope of cardiomyocytes [40]. It was first identified after expression of the truncated cDNA that encoded a 100-kDa fragment (this is why it was first called AKAP100) [32,41].

The mAKAP complex in the cardiomyocytes contains PKA, the cAMP-specific phosphodiesterase PDE4D3, PP2A, calcineurin, RyR2, ERK5, exchange protein activated by cAMP (Epac1) and other substrates of PKA [2,42]. Hence, AKAPs form a framework complex that synchronizes multi-protein signaling. Amino acids 2055–2072 of the mAKAP scaffolding protein are responsible for binding PKA-RIIα subunit and contain an important amphipathic α-helix. It has been also shown that a single amino acid change in this amphipathic region of (I2062P) causes a disruption of the helical structure and prevents the association of PKA to the scaffolding protein [32]. Additionally, mAKAP acts as a multi-signaling network protein by recruiting and coordinating PDE4D3 and compiling a negative feedback loop by assembling PDE4D3 and PKA at the perinuclear membrane [43,44]. PKA phosphorylation of PDE4D3 at Serine 13 increases the affinity of PDE4D3 for mAKAP, enhancing its recruitment and allowing for rapid signal termination through PKA phosphorylation of PDE4D3 at Ser 54; mAKAP binds to the *N*-terminus residues of PDE4D3, and PDE4D3 binds to the central residues 1286–1831 of mAKAP [45].

Besides calcineurin, adenylyl cyclase V (ACV) and Epac1, mAKAP can also bind PP2A, which upon being phosphorylated by PKA participates in PDE4D3 dephosphorylation [2,43]. This pattern provides all the required machinery for cAMP synthesis, function and degradation, which may cause local variation in cAMP and pulsation of localized PKA activity.

Furthermore, mAKAP is also an important scaffold for the Ca^2+^-mediated signaling near the perinuclear region. It is well established that RyR2 regulates muscle contraction via the release of Ca^2+^ from intracellular stores and governs cardiac excitation-contraction coupling. By scaffolding PKA near RyR2, mAKAP is able to facilitate phosphorylation of subset of RyR2 present on nuclear envelope. However, these subsets of mAKAP-bound RyR2 at the perinuclear region may not significantly alter cardiac contractility. A paper by Wu and Bers [46] explains that SR and nuclear envelope is interlinked to form a Ca^2+^ store that can maintain high luminal Ca^2+^. Also, PKA-mediated phosphorylation of RyR2 and its contribution in EC-coupling is highly controversial [47,48]. Hence, in light of these reports, we believe that mAKAP has little or no role in regulating EC-coupling of cardiomyocytes.

On the other hand, mAKAP plays a critical role in mediating excitation-transcription coupling via perinuclear Ca^2+^ signal transduction. It is reported that mAKAP is important for adrenergic mediated hypertrophy through the calcineurin-NFATc (nuclear factor of activated T-cells) pathway that is activated by the increase of Ca^2+^ release from RyR2. This will lead to calcineurin activation, which causes NFATc dephosphorylation and translocation into the nucleus, promoting the activity of several pro-hypertrophic genes leading to cardiac hypertrophy [49]. Additionally, it has been shown that reduced PDE4D3 levels in the RyR2 complex of failing human hearts, as well as PDE4D3 inactivation in mice is associated with progressive cardiomyopathy, accelerated heart failure, and cardiac arrhythmias [50]. However, the complete molecular mechanisms that explain the correlation between mAKAP multi-protein signaling complex and heart diseases remain unclear.

The anchoring protein mAKAP also orchestrates cross talk between the cAMP and mitogen activated protein kinase (MAPK) signaling pathways through scaffolding various proteins regulating these two pathways. It has been shown, using immunoprecipitation studies, that mAKAP is able to bind ERK5, which phosphorylates PDE4D3 at serine 579 causing a reduction of PDE activity. Thus, mAKAP organizes the bidirectional regulation of both PKA (the stimulator) and ERK5 (the inhibitor) of PDE activity [21].

In cardiomyocytes, mAKAP functions to regulate cAMP, to modulate substrate phosphorylation and to dephosphorylate proteins that are involved in cardiac function. Recently, there has been increasing evidence that shows that nuclear Ca^2+^ pool signals differently than cytoplasmic Ca^2+^ pool. Nuclear Ca^2+^ regulates expression of hypertrophic genes whereas cytoplasmic Ca^2+^ is essential for cardiac contractility [51]. Positioned at nuclear envelope, mAKAP-mediated signaling pathways also function as a gatekeeper for transcription factors. Considering the many crucial roles of mAKAP, aberrant PKA-mAKAP or PDE4D3-mAKAP anchoring may be the cause for some heart diseases [30,43,45].

## 3. Cardiac mAKAP and Hypertrophy Signaling

The common phenotype associated with heart failure is the development of cardiac hypertrophy. Hypertrophy is defined as physiological increase in heart size in order to compensate the increase in the work load. The heart is considered to be a highly adaptable organ that, upon either overt persistent increase in cardiac work burden or upon loss of proper cardiac blood supply, is able to compensate by increasing sympathetic nervous system (SNS) stimulation and activation of adrenergic receptors in order to increase cardiac output. In the acute setting, these adaptive mechanisms may save the heart, however chronic stimulation of the heart along with limited capacity reserve will cause extensive morphological changes and exacerbate cardiac dysfunction.

The persistent activation of kinases and phosphatases such as PKA, PKC, and calcineurin (PP2B) as a result of chronic β-AR stimulation leads to the activation of pro-hypertrophic genes. The activation of these genes triggers cardiac remodeling, which is characterized by changes in the cellular structure as well as in the size and shape of the heart. For instance, decreased PKA-dependent phosphorylation of cTnI, cMyBPC and PLB in heart failure has been linked to increase ventricular remodeling. Calcineurin dephosphorylates the transcription factor NFATc causing its translocation into the nucleus. Once in the nucleus, NFATc causes the transcription of genes involved in cardiac remodeling and hypertrophy [21,52,53].

mAKAP along with various other AKAPs have been associated with the induction of hypertrophy as they are involved in mediating signaling that initiates hypertrophic gene expression mainly through activation of the MAPK pathway [54,55]. For instance, some studies have shown that both adrenergic and cytokine induced hypertrophy can be reduced by downregulation of mAKAP [15,53]. Activation of the cytokine gp130 receptors using leukemia inhibitory factor (LIF) triggers hypertrophy via the ERK5 signaling pathway. The LIF-induced myocytes hypertrophy has been shown to be inhibited by: (1) displacement of the mAKAP from the perinuclear envelope through targeting nesprin-1α; and (2) gene silencing of mAKAP using siRNA. These data suggest that mAKAP expression is necessary for LIF-induced hypertrophy [21]. Additionally, a study done by Puckelwartz *et al.*, 2010 [56] revealed that a human missense mutation R374H, found within the mAKAP nesprin-1 alpha domain (a protein responsible for tethering mAKAP to the nuclear membrane) from a patient with dilated cardiomyopathy, required cardiac transplantation.

The scaffolding protein mAKAP is required for adrenergic agonist-induced cardiac hypertrophy through activation of the calcineurin/NFATc pathway. Hence, mAKAP should bind PKA for activation of myocyte hypertrophy through adrenergic agonist pathway [53]. Besides, mAKAP binds to phospholipase Cε (PLCε) which utilizes phosphatidylinositol 4-phosphate (PI4P) from Golgi apparatus near nuclear envelope to produce diacylglycerol (DAG). mAKAP bound protein kinase Cε (PKCε) in turn activates protein kinase D (PKD) to regulate hypertrophic signaling [57]. More recently, Kapiloff’s group have created cardiac-specific mAKAPβ knock out (KO) mice by conditional deletion of exon 9 [33]. In this paper, they added two new binding partners of mAKAP, PKD1 and HDAC4. In pressure-overload model of hypertrophy induced by transverse aortic constriction (TAC), mAKAPβ KO mice had improved cardiac status as compared to their WT littermates. Decreased phosphorylation of PKD1 and HDAC4, suppression of NFATc and MEF2 mediated expression of pro-hypertrophic genes were correlated with better cardiac health in mAKAPβ KO mice [33].

For the purpose of studying the human mAKAP structure–function relationship along with its physiological consequences in changing downstream signaling, *in vitro* pull-down assays, PKA and PDE activities, Ca^2+^ signaling and surface plasmon resonance experiments were studied by McConnell’s group [39]. After screening numerous non-synonymous polymorphisms available in the dbSNP databases (NCBI), University of California Santa Cruz (UCSC) and Ensemble Genome Browser, three different mutations of the human mAKAP protein, (1) P1400S (mutation in the PDE4D3 binding site); (2) S2195F (mutation in the PP2A binding site and also flanking the 3'-PKA-RIIα) and (3) L717V (mutation surrounding the spectrin repeat domain which anchors mAKAP to the perinuclear membrane) were chosen by McConnell’s team based on their Evolutionary Trace rank (where low values mean a greater potential impact) and the mAKAP domain location in which these mutations occur [39]. The immunoprecipitation results showed that mAKAP-S2195F (mutation in the PP2A binding site and also flanking the 3'-PKA-RIIα) mutant promotes a significant increase in binding affinity to PKA-RIIα. This serine is predicted to have a strong evolutionary importance and its replacement by a large and hydrophobic phenylalanine is likely to impact function. Indeed, this substitution increases mAKAP binding to PKA-RIIα, possibly due to increase in the number of hydrophobic amino acids in the hydrophobic ridge. This increase in PKA-RIIα binding affinity causes a significant increase in PKA activity, which explains the increase in Ca^2+^ release, calcineurin (PP2B) activity and CREB phosphorylation. The observed increases in these parameters suggest a possible induction of pro-hypertrophic gene expression. On the other hand, the other two mutations studied (mAKAP-P1400S and mAKAP-L717V) showed no significant difference from wild type in PKA activity although they showed a deviated binding propensity to PDE4D3 from wild type [39]. Collectively, these data suggest a model in which mAKAP acts as a main organizer of cAMP, Ca^2+^ and MAPK signaling and works to regulate cardiac hypertrophy, and thus with the established importance of mAKAP in cardiac hypertrophy, it is now critical to delineate the functional significance of known mAKAP SNPs and screen for additional mAKAP polymorphisms.

## 4. Conclusions

In conclusion, the role of protein kinases compartmentalization is critical in mediating the kinase signaling pathway and explains different disease pathologies’ development in the absence of these AKAPs. In particular, mAKAP network disruption provides an important role in cardiac hypertrophy signaling, and this may open a new avenue for more profound genetic screening and development of specific peptide inhibitors targeting drugs.
